# Molecular characteristics of clinical IMP-producing *Klebsiella pneumoniae* isolates: novel IMP-90 and integron In*2147*

**DOI:** 10.1186/s12941-023-00588-w

**Published:** 2023-05-15

**Authors:** Liuyang Yang, Guangcun Zhang, Qiang Zhao, Ling Guo, Jiyong Yang

**Affiliations:** Laboratory Medicine Department, First Medical Center of Chinese PLA General Hospital, Beijing, 100853 China

**Keywords:** Carbapenemase, Metallo-β-lactamases, IMP, *Klebsiella pneumoniae*, Integron

## Abstract

**Background:**

Since the first report of carbapenem-resistant *Klebsiella pneumoniae* isolates in China in 2007, the prevalence of CRKP and CRE has increased significantly. However, the molecular characteristics of IMP-producing *Klebsiella pneumoniae* (IMPKp) are rarely reported.

**Methods:**

A total of 29 IMPKp isolates were collected from a Chinese tertiary hospital from 2011 to 2017. Clinical IMPKp were identified by VITEK^®^MS, and further analyzed by whole-genome DNA sequencing with HiSeq and PacBio RSII sequencer. Sequencing data were analyzed using CSI Phylogeny 1.4, Resfinder, PlasmidFinder and the MLST tool provided by the Centre for Genomic Epidemiology. The analysis results were visualized using iTOL editor v1_1. The open reading frames and pseudogenes were predicted using RAST 2.0 combined with BLASTP/BLASTN searches against the RefSeq database. The databases CARD, ResFinder, ISfinder, and INTEGRALL were performed for annotation of the resistance genes, mobile elements, and other features. The types of *bla*_IMP_ in clinical isolates were determined by BIGSdb-Pasteur. Integrons were drawn by Snapgene, and the gene organization diagrams were drawn by Inkscape 0.48.1.

**Results:**

Four novel ST type, including ST5422, ST5423, ST5426 and ST5427 were identified. The IMP-4 and IMP-1 were the dominant IMP type. The majority of *bla*_IMP_-carrying plasmids belonged to IncN and IncHI5. Two novel *bla*_IMP_-carrying integrons (In*2146* and In*2147*) were uncovered. A novel variant *bla*_IMP-90_ presented in novel integron In*2147* has been identified.

**Conclusions:**

IMPKp showed low prevalence in China. Novel molecular characteristics of IMPKp have been identified. Continuous monitoring of IMPKp shall also be carried out in the future.

**Supplementary Information:**

The online version contains supplementary material available at 10.1186/s12941-023-00588-w.

## Introduction

The spread of carbapenem-resistant *Enterobacteriaceae* (CRE) has become a major public health problem. Carbapenems are the most effective drugs for the treatment of Gram-negative bacterial infections. In China, since the first report of carbapenem-resistant *Klebsiella pneumoniae* (CRKP) isolates in 2007, the prevalence of CRKP and CRE has increased significantly [1].

Resistance to carbapenems involves multiple mechanisms, and the production of various carbapenemases is the most common mechanism. Based on their dependency on actions for enzyme activity, carbapenemases can be divided into two different groups: serine/non-metallo- (zinc-independent; classes A, C, and D) and metallo-carbapenemases (MBLs; zinc-dependent; class B) [2]. MBLs include various clinically and epidemiologically important enzymes, such as IMP, VIM and NDM. Since the first report of IMP-1 from *Pseudomonas aeruginosa* in Japan, IMP-type enzymes have been reported globally. Compared with NDM, IMP exhibited a relatively low prevalence among clinical *K*. *pneumoniae* isolates [3]. The CRKP harboring the *bla*_IMP-1_, *bla*_IMP-4_, *bla*_IMP-22_ and *bla*_IMP-26_ have been recovered from East Asia, Europe, Australia, or North America [3]. In China, the main IMP variants include IMP-4, IMP-8, and IMP-26 [1].

The plasmid-mediated dissemination of carbapenemase-coding genes plays an important role in emergence and spread of CRKP. The most encountered *bla*_IMP_ genes located in plasmids with diverse incompatible groups include HI2/HI5, FI/FII, L/M, A/C, P and N [4]. Various integrons that contain *bla*_IMP_ genes had been characterized such as In*1763*, In*809*/In*823*, In*722*, In*73* and In*687*, and these integrons harbored *bla*_IMP-1_, *bla*_IMP-4_, *bla*_IMP-6_, *bla*_IMP-8_ and *bla*_IMP-14_, respectively [5, 6].

Few studies have reported the complete sequence of *bla*_IMP_-harbouring plasmids, which limited our understanding of the transmission mechanism of *bla*_IMP_ genes. In this study, 29 clinical IMP-producing *K. pneumoniae* (IMPKp) isolates were analyzed by whole-genome sequencing and further molecular analysis. A novel IMP-type enzyme (IMP-90) and novel *bla*_IMP_-harbouring integron (In*2147*) were identified.

## Material and methods

### Bacterial strains and antimicrobial susceptibility testing

A total of 29 IMPKp isolates were collected from July 2011 to November 2017 from our hospital (Additional file 1: Table S1). All isolates were identified by VITEK^®^MS (bioMérieux SA, Marcy-l’Étoile, France). At first, the MICs of antimicrobial agents [ceftazidime (CAZ), cefepime (CFP), ceftazidime-avibactam (CAZ-AVI), imipenem (IPM), meropenem (MEM), ertapenem (ETP), amikacin (AK), ciprofloxacin (CIP) and sulfamethoxazole/trimethoprim (SXT)] were measured by BioMérieux VITEK 2 AST-GN09 and AST-GN13 according to the manufacturer’s instructions. Then, were measured with broth microdilution method by using the Biofosun R Gram-negative panels (Biofosun Biotechnology Corporation Ltd., Shanghai, China). If the MICs of carbapenem antibiotics exceeded the upper limit of detection [IPM (MICs > 8 mg/L), ETP (MICs > 8 mg/L) or MEM (MICs > 4 mg/L)], the dry strip method was performed to correct the results by using E-test (Autobio Diagnostics Corporation Ltd., Zhengzhou, China). All susceptibility results were interpreted according to the 2021 CLSI guidelines [7].

### Whole-genome DNA sequencing

All isolates were subjected to draft-genome sequencing using a paired-end library with an average insert size of 350 bp (ranged from 150 to 600 bp) on a HiSeq sequencer (Illumina, CA, USA). All sequences were assembled using SOAPdenovo (SOAP Version 2.21). The N_50_, N_90_, coverage rate and scaffold number were used to identify de novo characteristics. In addition, one or two strains from each subclade were selected for subsequent long PacBio reads sequencing using a sheared DNA library with average size of 15 kb (ranged from 10 to 20 kb) on a PacBio RSII sequencer (Pacific Biosciences, Menlo Park, CA, United States). The paired-end short Illumina reads were used to correct the long PacBio reads utilizing proofreads, and then the corrected PacBio reads were assembled de novo utilizing SMARTdenovo.

### Data analysis

WGS data for all isolates were analyzed using CSI Phylogeny 1.4, Resfinder, PlasmidFinder and the MLST tool provided by the Centre for Genomic Epidemiology (https://cge.cbs.dtu.dk/services). Determination of capsular type for strains was conducted by using Kleborate (https://github.com/katholt/Kleborate). The tree file was visualized by iTOL (https://itol.embl.de), and annotated information were edited by iTOL editor v1_1.

### Sequence annotation

The open reading frames and pseudogenes were predicted using RAST 2.0 combined with BLASTP/BLASTN searches against the RefSeq database as previous described [8]. Plasmid replicon types were identified using PlasmidFinder and corrected by the RAST 2.0. The databases CARD, ResFinder, ISfinder, and INTEGRALL were performed for annotation of the resistance genes, mobile elements, and other features. The types of *bla*_IMP_ in clinical isolates were determined by BIGSdb-Pasteur. Integrons were drawn by Snapgene, and the gene organization diagrams were drawn by Inkscape 0.48.1.

### Transformation experiments

As previously described [9], we cloned the open reading frame of *bla*_IMP-90_ and *bla*_IMP-8_ into the chloramphenicol-resistant pHSG398 vector (Takara Bio, Shiga, Japan) at the *Eco*RI-*Kpn*I site and used it to transform *Escherichia coli* DH5α cells (Takara Bio).

### Nucleotide sequence accession numbers

All genome sequences in this study were submitted to GenBank under the accession BioProject No. PRJNA862666.

## Results

### Clinical characteristics of IMPKp isolates

**Fig. 1:**
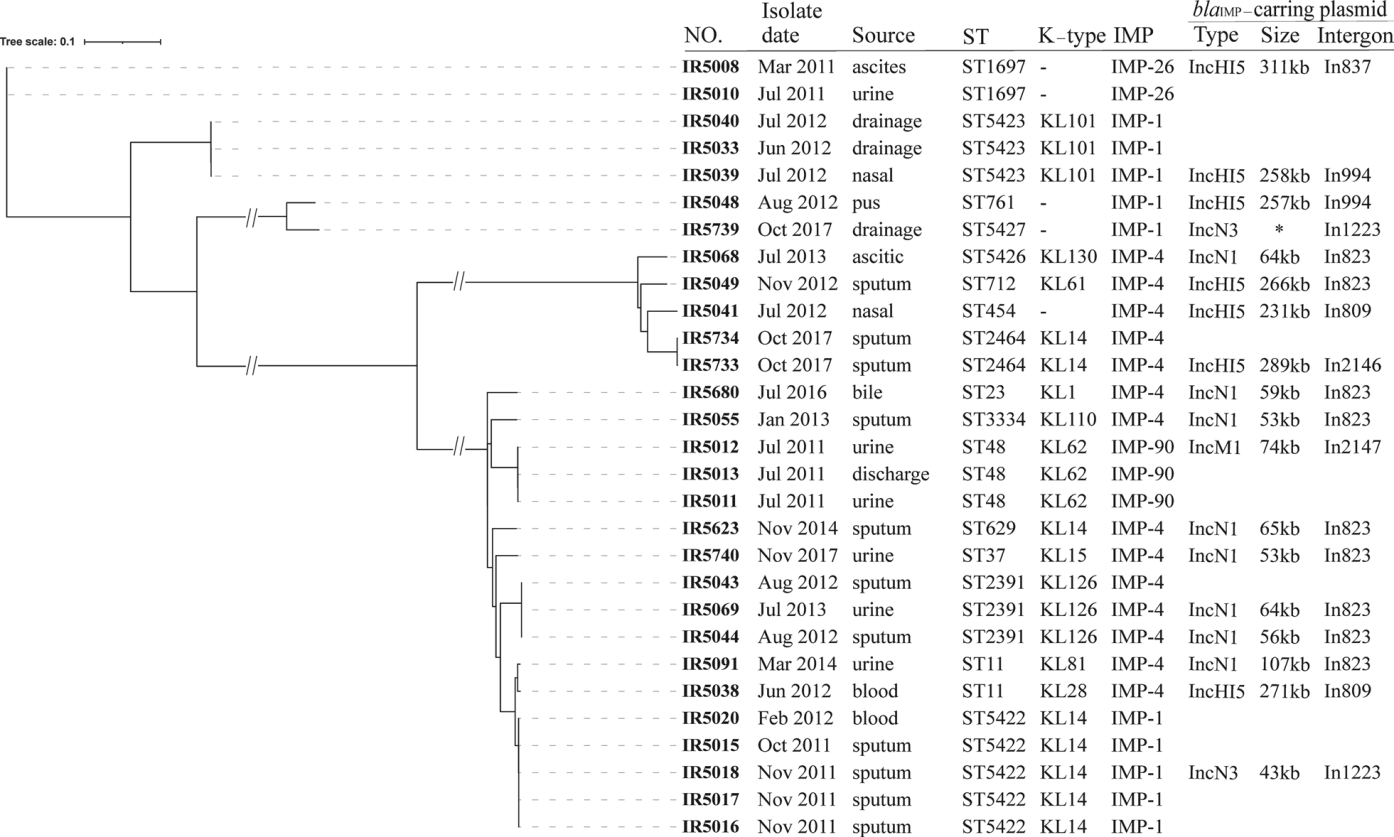
Genetic characteristics of IMP-producing *K. pneumoniae*. All the *bla*_IMP_-carrying plasmid were analyzed by the HiSeq + PacBio RSII, except that in IR5739 isolates was identified by HiSeq (marked with asterisks)

A total of 29 IMPKp isolates were collected from July 2011 to November 2017 (Fig. 1). The sputum (n = 11, 37.9%) and urine (n = 6, 20.7%) were the main sources of these isolates. The strains were mainly isolated from the departments of hepatobiliary (n = 9), neurology (n = 6), surgical care unit (n = 4) and respiratory medicine (n = 4). In this study, four novel STs (ST5422, ST5423, ST5426 and ST5427) with the allelic profile (*gapA, infB, mdh, pgi, phoE, rpoB,* and *tonB*) 3-3-1-1-1-1-9, 42-22-25-96-115-20-712, 16-24-21-31-47-257-760 and 17-73-90-39-541-18-148, respectively, have been identified. The ST5422 (n = 5), ST5423 (n = 3), ST48 (n = 3), ST2391 (n = 3) outbreaks may occur in our hospital (Fig. 1, Additional file 1: Table S1). The antimicrobial susceptibility results were listed in Table S1. About 55.2% (n = 16) of IMPKp isolates carried various *bla*_CTX-M_, while nine isolates (24.1%) carried *armA* which was related to the amikacin resistance.

### ***The plasmids and integrons related to known bla***_***IMP***_

The complete genome sequences of eighteen strains were analyzed. All *bla*_IMP_ gnes were located on the plasmids (Fig. 1 and Additional file 1: Table S1). In total, three known *bla*_IMP_ variants have been identified, including *bla*_IMP-1_, *bla*_IMP-4_ and *bla*_IMP-26_. The gene *bla*_IMP-4_ was distributed in 10 different ST types. Besides the ST761, other isolates harboring the *bla*_IMP-1_ were novel ST types, including ST5422, ST5423, and ST5427 (Fig. 1). The *bla*_IMP-1_-carrying plasmids belonged to IncN3 and IncHI5 with a size ranging from 43 to 258 kb. Plasmids harboring *bla*_IMP-4_ were IncN1 and IncHI5 with a size ranging from 53 to 289 kb. The *bla*_IMP-26_ located on a 311 kb IncHI5 plasmid (Fig. 1 and Additional file 1: Table S1). All *bla*_IMP_-carrying plasmids carried various resistance genes (Additional file 1: Table S1).

**Fig. 2:**
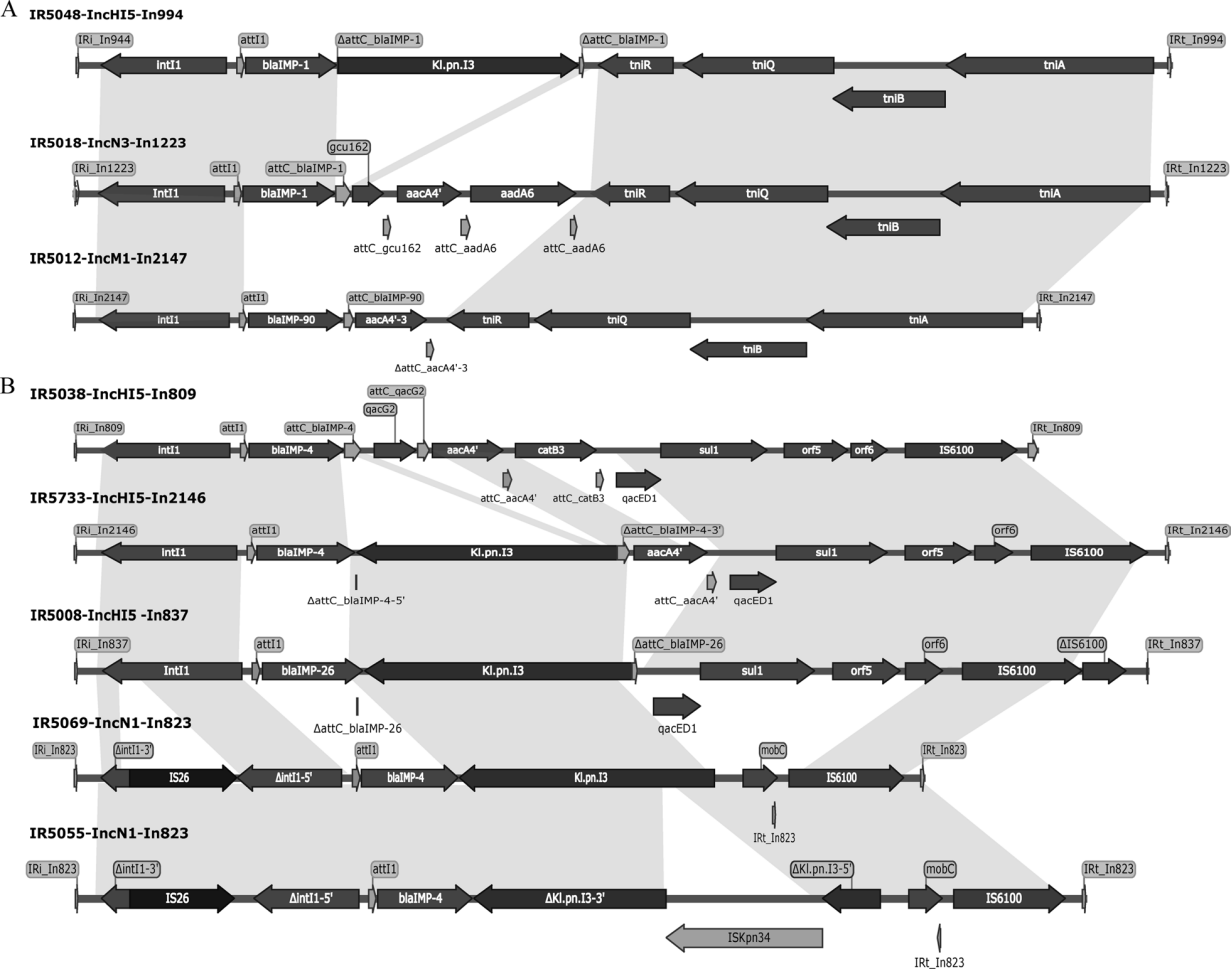
Comparative analysis of the *bla*_IMP_-carrying integrons. **A** The In*994*, In*1223* and In*2147* shown the similarity. **B** The In*809*, In*2146*, In*837* and In*823* shown the similarity

The *bla*_IMP-1_ was presented in the In*994* and In*1223*, the *bla*_IMP-4_ was presented in the In*809*, In*823*, and novel identified In*2146*, while the *bla*_IMP-26_ was presented in the In*837* (Fig. 2 and Additional file 1: Table S1). The integrons carrying the *bla*_IMP-4_ and *bla*_IMP-26_ exhibited a certain degree of similarity. Compared with the *bla*_IMP-4_-carrying In*809*, the novel In*2146* (*bla*_IMP-4_-*attCDΔ*::*Kl.pn.I3-aacA4'-attC-3'CS*) seems like inserted the *Kl.pn.I3* and lost the resistance genes *qacG2* and *catB3*. Meanwhile, there was a possibility that the In*2146* was derived from the In*823* by inserting the *aacA4* and *bla*_IMP-4_ mutation (Fig. 2). The In*809*, In*823* and In*2146* contained 3'-conserved segment (CS) structures immediately downstream of the gene cassettes. The majority contained In*4*-like structures consisting of 3'-CS-IS*6100* (with or without partial deletion of 3'-CS and *chrA*-*padR* insertion).

### Novel metallo-β-lactamase IMP-90 and integron In2147

**Fig. 3:**
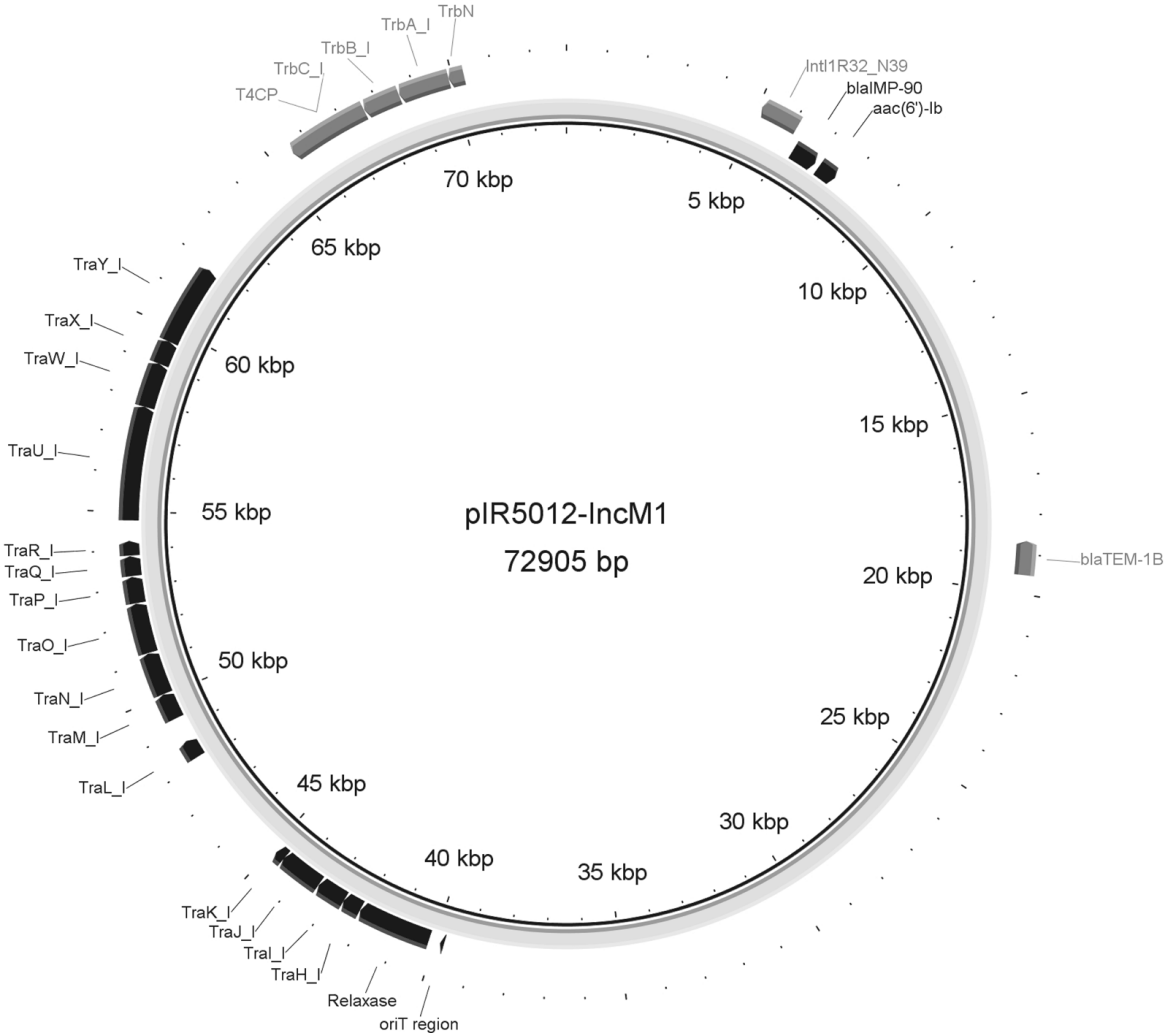
Backbone structure of the *bla*_IMP-90_-encoding plasmid pIMP-90-IR5012

In this study, a novel IMP-type MBL, *bla*_IMP-90_, was identified. An IMPKp ST48 emerged and caused an outbreak in 2011 (Fig. 1). Whole-genome sequencing revealed that the isolate carried a novel *bla*_IMP_ variant, which was designated as the *bla*_IMP-90_. IMP-90 differed from IMP-8 by an amino acid substitution (Ser262Gly). The *bla*_IMP-90_ was located on a 72905-bp IncM1 plasmid (Fig. 3), and was presented in a novel integron In*2147* (*bla*_IMP-90_*-attC-aacA4'-3-attCD*), which showed similarity with In*994* and In*1223* (Fig. 2). The IR5012 was susceptible to imipenem (MIC = 1 ug/mL), suggesting that the IMP-90 had a weaker imipenem hydrolyzing activity compared with other IMP enzymes (Additional file 1: Table S1). Meanwhile, the transformation experiments of *bla*_IMP-90_ and *bla*_IMP-8_ demonstrated that the IMP-90 exhibited similar carbapenem hydrolyzing activity with IMP-8.

## Discussion

The prevalence of IMPKp varies by regions and often induces outbreaks in clinical departments. A multicenter study showed that the MBLs were not equally common, with more than 60% of MBL-positive CRE isolates carrying the *bla*_NDM_, while only 9% of the isolates produced the IMP [3]. In China, the predominance of MBL among *Enterobacteriaceae* was NDMs, and the IMP has been sporadically detected [10]. We have reported the molecular characterization of some clinical IMPKp isolates [11]. In this study, a larger collection (n = 29) of IMPKp were collected and analyzed by more accurate methods such as whole-genome sequencing, and thus limited results in previous study are revised and supplemented. The high homology among some strains indicated the existence of small-scale outbreaks caused by various IMPKp clones (Fig. 1). Most IMPKp isolates exhibited low-level resistance or even susceptible to various carbapenems (Additional file 1: Table S1), indicating the low hydrolysis capacity of IMP, and may leading to a low epidemic level of IMPKp. In this study, IMP-4 (14/29, 48.28%) was the most popular enzyme type, followed by IMP-1 (10/29, 34.48%), and this was consistent with previous finding [12].

The ST type of IMPKp isolates in this study was relatively dispersed (Fig. 1). The *K. pneumoniae* CG258 are the most common carbapenem-resistant isolates reported worldwide, including the ST11 and the ST5422 in this study. The *tonB* allele of ST11 (*tonB*-4) is widely distributed in approximately 80 unrelated STs of CG258, but the *tonB*-79 allele of ST258 has only been observed among less than 10 STs (http://www.pasteur.fr/mlst). Some researchers considered that the *tonB*-79 was probably derived from *tonB*-4 by acquisition of site substitutions [13]. In this study, among the four novel STs (ST5422, ST5423, ST5426 and ST5427), only ST5422 [allelic profile (*gapA, infB, mdh, pgi, phoE, rpoB,* and *tonB*) was 3-3-1-1-1-1-9] may arise from ST11 (allelic profile: 3-3-1-1-1-1-4) by substituting *tonB*-4 with *tonB*-9. However, although it has high homology with ST11, completely different characteristics of IMP-coding genes suggested the independent evolutionary path of this novel identified clone (Fig. 1).

Consistent with previous study [14], we found that all known IMP-coding genes were located on IncN and IncHI5 plasmids in this study (Fig. 1). IncN and IncHI plasmids are both conjugative plasmids and have been reported to be part of a broad-host-range group [15]. Based on the nucleotide sequence homology over the backbones, the IncN group can be divided into three subgroups, including IncN1, IncN2 and IncN3 [16]. In this study, the predominant *bla*_IMP_-carrying plasmids belonged to N1 with a smaller size (Fig. 1). The *bla*_IMP-1_-carrying IncN3 plasmid was identified in UK [5], and this was the first report to identify this plasmid in China, showing the necessity of investigating prevalence and evolutionary history of IncN3 plasmids. The IncHI plasmids are important vectors in the dissemination of heavy metal resistance genes and antimicrobial resistance genes, and usually have a size larger than 200 kb [17]. In this study, all *bla*_IMP_-carrying IncHI5 plasmids carried conserved IncHI5 backbones, including *repHI5B* and a *repFIB*-like, *parABC*, and *tra1*, mediating replication, partition and conjugal transfer, respectively. Further studies are needed to continuously monitor the prevalence of *bla*_IMP_-carrying IncN and IncHI5 plasmids in different sources, especially among clinical settings [11, 14].

To date, various genetic context of *bla*_IMP-4_ has been identified, e.g. *bla*_IMP-4_*-qacG-aacA4-aphA15, bla*_IMP-4_*-Kl.pn.I3-mobC and bla*_IMP-4_*-Kl.pn.I3-qacEΔ-sul1*. Of note, the structure of *bla*_IMP-4_*-Kl.pn.I3* seems unique in isolates from China revealed by blasting in GenBank, thus it could be used as an epidemiological marker for *bla*_IMP-4_ detection in China. In*823* harboring *bla*_IMP-4_ has been identified in IncHI5 plasmid [18]. In this study, all In*823* harboring the *bla*_IMP-4_ was presented in the IncN1 plasmid, and the *bla*_IMP-4_*-attCDΔ*::*Kl.pn.I3* was the most common cassette. Meanwhile, the integrons in IncHI5 were In*809* and the novel integron In*2146* (Figs. 1 and 2). In*809* with four cassettes (*bla*_IMP-4_-*qacG2-aacA4-catB3*) is widely disseminated in *Enterobacteriaceae* and *Acinetobacter* spp. in Asia–pacific region [19]. In*2146* may be derived from In*809*, and is considered as an In*809*-like integron (*bla*_IMP-4_*-Kl.pn.I3-qacG2-aacA4-catB3Δ*), of which a group II intron *Kl.pn.I3* was inserted into the *attC* site of the *bla*_IMP-4_ cassette (Fig. 2). The *bla*_IMP-1_-carrying In*994* and In*1223* has been identified in previous studies [16], and it has also been found in this study (Fig. 1). In addition, the integron In*837* (*intI1-bla*_IMP-26_-*att*CDΔ::*Kl.pn.*I3-3'CS, Fig. 2) showed low homology with other *bla*_IMP-26_-carrying integrons, and had different genetic structure such as *intI1*-*bla*_IMP-26_-*ltrA-qacEΔ1-sul1*, *intI1-bla*_IMP-26_*-qacG-aacA4-aac(6ʹ)-orf-catB3*, and *intI1-bla*_IMP-26_*-qacG-aac(6ʹ)-Ib-aac(6ʹ)-orf3-orf4-catB3-dfrA1-tnpA-istB-orf5*, respectively [20]. In general, country-specific *bla*_IMP_ subtypes corresponded to the specific integron types previously characterized in that country, i.e., *bla*_IMP-4_-carrying In*809* in Australia [21]; *bla*_IMP-6_-carrying In*722* in Japan [22]; *bla*_IMP-8_-carrying In*73* in Philippines [23]; and *bla*_IMP-14_-carrying In*687* in Thailand [24]. In this study, we identified 7 different *bla*_IMP_-carrying integron types, including 2 novel cassette combinations (In*2146* and In*2147*; Fig. 2). All the four class I integrons have a complete set of IRi/IRt, *intI1*, and *attI1*. All integrons carried the strong promoters PcS (In*2147* and In*809*) or the PcW_TGN-10_ (In*837*, In*994*, In*823*, In*1223* and In*2146*) (Additional file 1: Table S1), and these promoters can drive the high-level expression of cassette-borne genes [25, 26].

IMP-90 is a novel variant with an amino acid substitution at Ser262Gly compared with IMP-8. The IMP-90-producing isolates showed a sensitive feature to imipenem (MIC = 1 mg/L), while the *bla*_IMP-90_-carrying *E. coli* DH-5α (competent cell) showed a reduced resistance to imipenem, compared with the *bla*_IMP-8_ (data not published). Previous studies show that isolates producing the IMP-type metallo-beta-lactamases with Ser262Gly substitution all exhibited susceptibility to imipenem, including the IMP-6 and IMP-68, which were the Ser262Gly substitution variants of IMP-1 and IMP-11, respectively [9, 27]. These results indicate that the Ser262Gly substitution may affect the hydrolysis ability of IMP variants to imipenem. The *bla*_IMP-90_ presented in the novel integron In*2147* located on a IncM1 plasmid (Figs. 2 and 3). Plasmids belonging to the IncL/M are the important mobile genetic platforms for dissemination of clinically important resistance genes, e.g. *bla*_CTX-M_ and *bla*_OXA-48_ [28]. Although L and M plasmids showed high level of DNA homology (approximately 94% overall nucleotide identity), the ExcA, TraY, and TraX proteins exhibited evident division (35%, 59%, and 75% amino acid identity, respectively). IncM plasmids have a wide host range, while IncM1 and IncM2 showed the 99% amino acid identity of the entry exclusion ExcA and TraY proteins [29]. This was the first report about the *bla*_IMP_-carrying IncM1 plasmid. Conjugation between isolates carrying various IncM plasmids is compromised by inhibitory interactions of the exclusion system, which is highly conserved across all IncM plasmids (Fig. 3) [29]. Therefore, more attention should be paid to the transmission of drug resistance mediated by the IncM-type plasmids, and continuous monitoring shall also be carried out.

## Conclusions

In conclusion, we collected and analyzed 29 IMPKp isolates from a Chinese tertiary hospital during the year 2011 to 2017. In this study, we identified four novel ST type, including the ST5422, ST5423, ST5426 and ST5427. The dominant IMP type was IMP-4 and IMP-1. The majority of *bla*_IMP_-carrying plasmids belonged to IncN and IncHI5. Two novel *bla*_IMP_-carrying integrons (In*2146* and In*2147*) were uncovered. A novel variant *bla*_IMP-90_ presented in novel integron In*2147* has been identified.

## Supplementary Information

**Additional file 1: Table S1.** Characteristics of 29 clinical *bla*_IMP_-carrying *Klebsiella pneumonia* isolates.

## Data Availability

All genome sequences in this study were submitted to GenBank under the accession BioProject No. PRJNA862666.

## Abbreviations

IMPKp: IMP-producing *Klebsiella pneumoniae*
CRE: Carbapenem-resistant* Enterobacteriaceae*
CRKP: Carbapenem-resistant *Klebsiella pneumoniae*
Inc: Incompatibility
RAST: Rapid Annotation using Subsystem Technology
CLSI: Clinical and Laboratory Standards Institute
MLST: Multi-locus sequence typing
MDR: Multi-drug resistance
IRi: Inverted repeat at the integrase end
CS: Conserved segment
IRt: Inverted repeat at the *tni* end
*E. coli*: *Escherichia coli*
MIC: Minimal inhibit concentration
CAZ: Ceftazidime,
CFP: Cefepime
CAZ-AVI: Ceftazidime-avibactam
IPM: Imipenem
MEM: Meropenem
ETP: Ertapenem
AK: Amikacin
CIP: Ciprofloxacin
SXT: Sulfamethoxazole/trimethoprim
